# Factors Associated with the Need for Assistance among the Elderly in Malaysia

**DOI:** 10.3390/ijerph18020730

**Published:** 2021-01-15

**Authors:** Nazarudin Safian, Shamsul Azhar Shah, Juliana Mansor, Zulkefley Mohammad, Siti Rohani Nurumal, Wan Abdul Hannan Wan Ibadullah, Saharuddin Ahmad, Yugo Shobugawa

**Affiliations:** 1 Department of Community Health, Faculty of Medicine, Universiti Kebangsaan Malaysia, Cheras 56000, Kuala Lumpur, Malaysia; nazarudin@ppukm.ukm.edu.my (N.S.); p102420@siswa.ukm.edu.my (J.M.); p102416@siswa.ukm.edu.my (Z.M.); p102422@siswa.ukm.edu.my (S.R.N.); p102417@siswa.ukm.edu.my (W.A.H.W.I.); 2 Department of Family Medicine, Faculty of Medicine, Universiti Kebangsaan Malaysia, Cheras 56000, Kuala Lumpur, Malaysia; saha@ppukm.ukm.edu.my; 3 Division of International Health (Public Health), Niigata University Graduate School of Medical and Dental Sciences, Niigata 950-2181, Japan; yugo@med.niigata-u.ac.jp; 4 Department of Active Ageing (Donated by Tokamachi City, Niigata, Japan), Niigata University Graduate School of Medical and Dental Sciences, Niigata 951-8510, Japan

**Keywords:** nursing care, need of assistance, elderly, old age

## Abstract

The need for assistance among the elderly is rising, which poses challenges for healthcare systems. Thus, this study aims to determine the factors associated with the need for assistance in the daily living activities of Malaysia’s elderly population. A total of 1204 elderly individuals, aged 60 years and above, were recruited. An interview was conducted using the Bahasa Malaysia version of the Japan Gerontological Evaluation Study (JAGES-BM) questionnaire. Overall, 7.7% of the elderly participants required assistance. A logistic regression model showed that difficult financial statuses (aOR 4.56), hearing difficulties (aOR 1.78), and severe limitations in daily activity over the past 6 months (aOR 11.99) were associated with a higher likelihood of needing assistance. In addition, daily activities such as an inability to feed (aOR 8.46), stand without support (aOR 2.06), or walk for 15 min without stopping (aOR 1.99) were significantly associated with the need for assistance. Factors associated with the need for assistance are health status, disability, and the financial status of the elderly. Preventive measures should be included in policies to ensure the sustainability of the care provided to the elderly in terms of promoting healthy ageing and a good quality of life.

## 1. Introduction

An elderly population is defined as people aged 65 years and older, and its proportion is increasing worldwide. The United Nations reported, in 2019, that 9% of the population are in this category, and this will increase to 16% by 2050 [1]. However, in some countries, the definition of elderly has been stated as being those who are 60 years and older [2]; therefore, the ageing population is becoming more prevalent in those countries [3,4]. In Malaysia, the same trend is observed as, within a year, the elderly population increased from 2.12 to 2.21 million between the third quarter of 2018 and the third quarter of 2019 [5]. The elderly population in Malaysia is expected to double, from 7.5% in 2020 to 15% in 2040 [6]; hence, the determining factors associated with the need for personal assistance is becoming important. Additionally, these are crucial details that have to be disclosed, especially among public health physicians, in order for them to be prepared with more structured program to give the best care to this population group later in their lives.

A change in population structure is expected because of prolonged life expectancy and concurrent fertility reduction [7]. Problems arise when the elderly have concomitant chronic diseases, as these are associated with health and economic burden [7,8,9]. Healthy ageing and a good supportive environment will allow the elderly to participate in the activities they value with minimal limitations. Therefore, healthy ageing is crucial for the elderly and, even when they have reduced capacity, a supportive environment is needed as it promotes dignity, autonomy, functioning, and continued personal growth [3]. Managing the need for personal assistance among the elderly allows them to be independent without ignoring the support they require.

Previous studies have shown a minimum of one activity of daily living (ADL) and one instrumental activity of daily living (IADL), such as shopping and housekeeping, by 1 million and 2.5 million elderlies, respectively, requires assistance [10]. Many of the activities that commonly require assistance involve mental health and well-being, and they include staying healthy, getting around, seeing, hearing, and communicating. These are associated with quality of life, level of dependency, risk of falls, and the need for assistance [11]. Nunes et al. further classified the needs of assistance into the following four categories: no need (requiring no caregiver), minimum need (requiring a caregiver sporadically), moderate need (requiring a caregiver intermittently), and maximum need (requiring a full-time caregiver). This classification system allows a person to identify the level of assistance an elderly individual requires and helps the caregiver tailor the needs of the elderly [12].

The Malaysian population comprises individuals with different races, which include Malays, Chinese and Indians, and different religions. The national or official language is Malay. This standard language is a symbol of unity across all ethnicities within the Malaysian population. Due to the ageing of the population, it is necessary to identify the factors associated with the need for assistance and the effect of daily living activities within this multiethnic population. This study will focus on sociodemographic, socioeconomic, and health statuses such as general health, physical and functional status that include types of difficulties, ADL, IADL, and mobility and falls.

## 2. Materials and Methods

### 2.1. Study Design

The Research Ethics Committee of the National University of Malaysia had approved this study (FF-2018-532). A cross-sectional study was conducted in Selangor, Malaysia from 1 December 2018 to 30 April 2020. Selangor is a state on the west coast of Peninsular Malaysia, encircling the capital Kuala Lumpur. Selangor was selected because it is the most populous state in the country, with a population of 6.53 million recorded in 2019 [13]. It represents a diversity of people and living conditions. Selangor has nine districts and 177 sub-districts. Among the Selangor districts, Hulu Langat, with a population of more than 1 million, and Kuala Selangor district, with a population of 0.2 million, were chosen. Those populations comprise all major ethnic groups such as Malay, Chinese, and Indian.

Official public data on administrative units and their population have been used as the sampling frame for clusters. Hulu Langat was selected as a representative of the urban area. Kuala Selangor was selected as a representative of the rural area. Multistage cluster sampling with probability proportionate to the size of the older population was conducted. The primary sampling unit (PSU) is the district, specifically Hulu Langat (urban) and Kuala Selangor (rural). The secondary sampling unit is the sub-district. Six sub-districts from Hulu Langat (which has seven sub-districts) and Kuala Selangor (which has nine sub-districts) were selected. At the third stage of sampling, ten towns/villages were randomly selected from each sub-district. A typical sub-district has about 30–50 towns/villages. The household ledgers for the selected areas were obtained with permission from the relevant village head, and they were used as the sampling frame for households and individuals. A random sampling of households that had an older person was conducted from the selected areas. A Kish grid table was used to select the sample when more than one elderly adult was eligible for the study in a selected household.

The sample size was calculated using the $n=Z^{2}P\left(1-P\right)/e^{2}$ equation in [14], where *Z* is level of confidence, *p* is the prevalence of ‘good health’ among older persons, and *e* is the margin of error. Using *Z* = 1.96, *p* = 0.3 (estimate obtained from a previous study conducted on older persons in Japan) [15], and *e* = 0.05, the initial calculation for sample size was 322. This initial sample size was then multiplied by the design effect of 1.5 and the two groups of estimates (urban and rural) desired for the survey results, giving a final figure of 966. Finally, 966 was divided by 0.80 to adjust for an anticipated 20% non–response rate, resulting in a total sample size of 1207.

Respondents were given a thorough explanation of the study, with information sheets and consent forms, before the interview. After the respondents signed the consent form, the interviews were conducted in a quiet environment, face-to-face, by trained research assistants, and it lasted 40–50 min. The study used the Bahasa Malaysia version of the Japan Gerontological Evaluation Study (BM-JAGES) questionnaire [16], which includes multi-dimensional variables. The variables comprised sociodemographic and socioeconomic characteristics, and health status included difficulties and daily living activities.

The inclusion criteria for the respondents were: (1) aged at least 60 years and the ability to converse in Malay or English; (2) registered residents of Malaysia (as household ledgers were used as the sampling frame); (3) living at home; and (4) able to understand the research and agrees to cooperate. However, the person was excluded if he/she was unable to cooperate and had an Abbreviated Mental Test (AMT) score of less than seven in the screening questions. A score of less than seven is considered to be an indication of severe cognitive function impairment. Those who were institutionalized in nursing or old folks’ homes were also excluded. The reason for excluding those with cognitive impairment was the difficulty in assessing the reliability and validity of their responses to the survey questions. Proxy respondents were also not considered for those excluded due to physical, mental, or cognitive impairment. There are no applicable guidelines for selecting an appropriate proxy (e.g., “legal surrogates” of older people are uncommonly designated in these country contexts). The comparability of responses obtained from the older person him/herself and those obtained from proxies would also pose a challenge for the data analysis and interpretation. Finally, we successfully recruited 1204 respondents with a response rate of 99.8%. Three persons were not recruited as they had an AMT score of less than seven.

### 2.2. Dependent Variables

The dependent variable in this study was the nursing care or assistance required by the respondent. It was based on the JAGES questionnaire “Do you need any nursing care or assistance in your daily life from anyone?” It was divided into the following two categories: ‘I do not need nursing care or assistance’ and ‘I need nursing care or assistance’. The classification was based on three initial answers for the need for assistance: ‘I do not need’, ‘I need but do not receive’, and ‘I need and receive’. Given the low proportion of respondents who answered ‘I need but do not receive’ and ‘I need and receive’ (2.1% and 5.6%, respectively), these two answers were then combined to become ‘I need nursing care or assistance’.

### 2.3. Covariates

The covariates were divided into the following three groups: (1) sociodemographic, such as the location of the respondent (lives in either rural or urban area), age group, gender, ethnicity, marital status, and family composition; (2) socioeconomic, such as financial and employment status; and (3) health status, such as general health, physical, and functional status, which in turn includes types of difficulties, ADL, IADL, and mobility and falls.

The age group was divided into three categories as follows: young elderly (aged 60–74 years), mid elderly (aged 70–84 years), and late elderly (aged 85 years and above). Ethnicity was divided into two groups, either Malay or non-Malay, given that most of the respondents were of Malay ethnicity. Marital status was divided into married, never been married, and divorced/widowed. The family composition was grouped into lives alone or lives with someone else, either family members or non-blood-related, including institutional bodies.

The financial status is more the perceived status rather than the real income that has been counted. This was divided into difficult, average, and comfortable. Employed, retired from a job, and never had a job constituted employment status. As part of the health status variables, Body Mass Index (BMI) classification was based on Malaysia’s BMI classification, which includes underweight (<18.5), normal (18.5–22.9), pre-obese (23.0–27.4), and obese (≥27.5) [17]. ‘Current health status’ was elicited with a self-declaration question: “How is your current health status?” The respondents selected the answer from three options, these being whether they are in a good, fair, or poor health. Types of difficulties that were assessed include seeing, hearing, mobility, and memory. The responses were divided into two categories, where “yes” meant they had the difficulty and “no” meant they did not have the difficulty.

In this study, we separated ADL and IADL from the rest of the covariates. The ADL that were assessed were bathing, dressing, toilet use, transferring and self-control of continence, and feeding. ADL responses were recorded as dichotomous categorical data (independent/dependent or good control/poor control). The IADLs that were assessed were being able to go out alone, being able to go shopping, being able to cook for themselves, being able to pay bills, being able to withdraw money, being able to fill out documents by themselves, and being able to find a friend’s telephone number. The responses for the IADLs were also recorded as dichotomous categorical data (yes/no). The following variables assessed mobility and fall: being able to go upstairs without holding on to handrails, being able to stand up from a chair without holding on to anything, being able to walk for 15 min without stopping, decreased frequency of going out, history of falls over the past year, and concern about falls. These responses were recorded as categorical data.

### 2.4. Data analysis

Data analyses were performed using IBM SPSS version 21.0 (IBM Corp., Armonk, NY, USA) with a *p*-value of less than 0.05 being considered significant in all tests. The chi-square test was used for bivariate analysis. Subsequently, the simple logistic regression and the forward likelihood ratio (LR) method of logistic regression was used for multivariable analysis. Interaction and multicollinearity were checked. All the covariates in the study were described in association with the dichotomous dependent variables: ‘I do not need nursing care or assistance’ and ‘I need nursing care or assistance’.

## 3. Results

Overall, only 8% of the respondents needed assistance and, out of those, 75% received the assistance they needed. The median age of all respondents was 68 years (interquartile (IQ) range, 63–72), and most of them were young elderly (83%). There were an equal distribution of respondents based on house location. Most of the respondents were male (57%) and of Malay ethnicity (83%). Sixty-six percent of respondents had an average financial situation, and only 14% were currently employed. A small percentage of the respondents (4%) had a poor health status; however, 41% claimed to have had limitations in daily activities within the past six months (Table 1). Concerning daily living activities, the most impaired ADL was self-control for continence (3%), while, for IADL, it was the ability to fill out a document by themselves (43%) (Table 2).

The bivariate analysis revealed that sociodemographic and socioeconomic variables, age group, and current financial status were the only variables significantly associated with the need for assistance among the elderly. Alternatively, current health status and all four types of difficulties, which are seeing, hearing, mobility, and memory or concentrating, were significantly associated with the need for assistance. Additionally, being ill for the past 12 months and having a limitation of activities within the past 6 months were significantly associated with the need for assistance (Table 1). Thus, ADL, IADL, mobility, and falls, but not concern about falls, were significantly associated with the need for assistance (Table 2).

Finally, the multivariable analysis noted that five factors: current health status, current financial status, hearing difficulties, mobility difficulties, and limitations in daily activities within the past 6 months, were associated with the need of assistance in the model (Table 3). The model correctly classified 92.3% of the respondents. Neither interaction nor collinearity was present. In Table 4, the need for assistance was associated with the ability to do the following by themselves: feeding, standing up from a chair without support, paying bills, and walking for 15 min without stopping, along with a history of falls in the past year. This model classified 92.5% of the respondents correctly.

Further analysis combining all the variables, sociodemographic and socioeconomic, and health status, including daily living activities, revealed that six covariates were significant (Table 5); current health status and mobility difficulty from Table 3, the ability to pay bills, and a history of falls (Table 4) were removed. The model correctly classified 92.8% of the respondents. A sub-analysis of elderly with a need for assistance revealed that the assistance was not associated with any factors or ADL, IADL, and fall and mobility variables.

## 4. Discussion

This study shows that most elderly individuals were dependent and did not need assistance. Nevertheless, none of those who needed assistance should be sidelined. The study provided a better understanding of the factors associated with the need for assistance among Malaysia’s elderly. Previous studies have identified five major groups of dependent-living factors: low functional capacity, poor health, personal attributes, resources, living circumstances, and social environment [18]. Almost all of these factors were covered in this study. The number of older people who needed nursing care or assistance in daily life activities was small, and of those, 2.1% did not receive it and 5.6% did. It is not easy to compare the percentage of need for assistance across studies due to considerable differences in study methodology and the definitions of variables used.

Nevertheless, as in this study, the low percentage for the need for assistance can be explained by the fact that most elderly individuals (59.1%) claimed to be in good health. Only 4.1% had experienced a severe limitation in activities over the past 6 months. However, a qualitative study noted that there was asynchronous behavior between subjective and objective health status, in which an elderly person might have overrated or underrated their health condition [19]. On the other hand, a study conducted in the United States showed that more than 58.5% of people aged between 85 and 89 received family assistance due to health problems or functional limitations [20]. Meanwhile, in Thailand, approximately 25.0% of the elderly have a certain degree of disability requiring support and assistance for ADLs [21].

Regarding the type of difficulty faced by the elderly, hearing difficulty and difficulty in walking or mobility were the only difficulties associated with the need for assistance. No difficulty in walking, climbing steps, and carrying items also negatively predicted the need for assistance for the elderly. Older people with no mobility difficulty tend to be physically active, have a lower risk of disability, and need no assistance. A study done in Finland revealed that older people who were physically active and without mobility impairments had a lower risk of dependency [22]. As for hearing difficulty, this can be easily managed with the use of a hearing aid. Hearing difficulties affect physical functioning, quality of life, cognitive functioning, and communication, and hence require assistance [23]. However, it is common for elderly individuals to have unperceived hearing difficulties, especially those with no previous working experience in a noisy environment [24]. Nevertheless, hearing difficulty is not associated with the need for assistance in some populations [11].

In terms of the limitation of physical activity in the past six months, elderly individuals that were severely limited were 12 times more likely to need assistance. This finding conforms to a study conducted in Gombak, Malaysia, in 2013, whose aim was to assess the self-reported physical activity among community-dwelling elderly. It was found that 58.1% of the included individuals reported being physically disabled, and the percentage was twice as high among mid elderly compared to young elderly [25]. Meanwhile, regarding Spanish elderly people, a Spanish National Health Survey that was carried out between 2009 and 2014 showed that 12.3% of the elderly population found it severely difficulty to walk 500 m as part of their physical activity without the need of assistance [26]. This difficulty showed some form of limitation in their physical activity, which would be better addressed with assistance in some form.

In terms of age groups, elderly status is further divided into young elderly, mid elderly, and late elderly. The older the person is, the more likely they need assistance in performing their ADLs. A study conducted in France, which included 8745 elderly people who were all more than 60 years old, showed that those who were more than 80 years old need assistance with at least one ADL [10]. Another study done in Israel yielded similar outcomes; among 1820 Israelis aged 75 to 94, the most dependent and needing assistance in at least one ADL were found to be those between the ages of 90 and 94 years [27].

Although other sociodemographic characteristics were not significant in this study, few other studies have shown a significant association. For instance, a cross-sectional survey done in the Philippines showed that women of older age, who were unmarried and living in urban areas with lower socioeconomic status, needed more assistance than their counterparts [28]. Meanwhile, in West Bengal, India, a study was done to compare daily living activities among the elderly aged 80 and above between rural and urban settings. Those who lived in urban areas were more likely to need assistance to perform ADLs [29]. A cohort study in China showed that those who needed assistance to carry out ADLs among older adults were higher among women who were aged more than 90 and lived in urban areas [30]. In addition, particularly in Asia, family members, including children and spouses, remain as a key provider and assume roles in taking care of older adults [31], similar to the situation found in the current study. The support could be in the form of co-residence, financial support, assistance in performing ADLs, or emotional and social support [32]. This culture is being practiced largely among Asian countries and is motivated by the concept of filial piety. The elderly should be respected, and it is the children’s and spouses’ responsibility to take care of their parents, who have sacrificed so much to raise them [33]. This is the reason why most elderly people live with family members.

In terms of socioeconomic status, those with a difficult financial status were 4.6 times more likely to be in need of assistance than those who were comfortable. Hypotheses regarding the connection between financial status and well-being center around three mechanisms [34,35,36,37]. The first is a realist one; those with higher livelihoods can buy better food, better lodgings, live in more secure conditions, and have better access to medical services. The second underlines conduct or “way of life” factors, for example, smoking, diet, liquor utilization, and suitable utilization of medical services, which change with intellectual ability and admittance of data. The third concentrates on psychosocial factors, such as strengthening, relative economic well-being, and social incorporation, including an introduction to stress that result from low status and self-governance in significant fields of life (e.g., work). This subsequently reduces the dependency of nursing care or assistance among the elderly population who have a good financial status.

In this study, most respondents (95.3%) were independent in terms of ADL. On the other hand, a study in China by Zhang L. et al. showed that 66.9% of their study population had no ADL limitations [38]. In our study, those who depend on all measured ADLs, IADLs, mobility, and fall showed a significant association with the need for assistance. However, Zhang L. et al. revealed that personal care and shopping activities were not significantly associated with the need for assistance. In our study, feeding dependency predicted the need for assistance, although the prevalence was too small (0.4%). Comparatively, a study done in the United States showed that 6.2% of the study population needed assistance for feeding [39]. Another study focusing on self-feeding dependency showed that older people admitted to a nursing home with slight feeding difficulty would need frequent and long-term assistance [40]. The study also suggested that most nursing home residents required feeding supervision after six months.

The IADLs are activities that require more complex thinking skills, including managing abilities. In this study, the IADL variable ‘inability to pay bills’ positively predicted the need for assistance. This variable assesses the complex skill of managing finances. The ‘inability to go upstairs without holding on to the handrail or the wall’, ‘inability to walk for 15 min without stopping’, and ‘history of falls’, which are related to mobility and falls, positively predicted the need for assistance. However, it can be argued that these two variables come in the ambulating part of ADL, as mentioned by Edemekong et al. [41]. Nevertheless, when comparing ADLs and IADLs, the risk of the need for assistance is higher when ADLs are affected. This finding was based on a prospective cohort study that assessed disability in older people by Bleijenberg N. et al. [42]. The authors showed that the impairment of IADLs had a low risk of general disability and did not increase with age.

As observed, this study has its limitations. Given the different population characteristics in different scenarios, the research findings may not be compared to others in all situations. However, because our tool has been validated within the population of interest and the samples obtained represent Malaysia’s population, the findings of this study are considered valid in regards to the multiracial population. Moreover, most of the variables were based on the individual’s perception rather than the true condition based on a certain diagnostic method.

## 5. Conclusions

In conclusion, the factors associated with the need for assistance among elderly individuals in Malaysia are their health status, disability, and financial status. Besides that, mobility and falls were more associated with the need for assistance than ADLs or IADLs. Although, at the moment, the percentage for the need of assistance was not high, in the future, as the population ages, it is expected that the demand for nursing care or assistance for the elderly population will increase. Therefore, with the identification of groups of elderly that need assistance the most, preventive measures should be included in policies and be made known to the public. This will ensure the sustainability of the care provided to the elderly and promote healthy ageing and a good quality of life for all elderly people.

## Figures and Tables

**Table 1:** Descriptive and bivariate analysis of factors associated with the need for assistance.

Variables	Total, *n* (%)	I Do not Need Assistance, *n* (%)	I Need Assistance, *n* (%)	^a^*p*-Value
Locality				
Rural	602 (50.0)	552 (91.7)	50 (8.3)	0.45
Urban	602 (50.0)	559 (92.9)	43 (7.1)	
Age	68 (63–72) *	N/a	N/a	N/a
Age group				
Young elderly	996 (82.7)	929 (93.3)	67 (6.7)	0.018
Mid elderly	186 (15.4)	163 (87.6)	23 (12.4)	
Late elderly	22 (1.8)	19 (8.6)	3 (1.4)	
Gender				
Male	691 (57.4)	640 (92.6)	51 (7.4)	0.604
Female	513 (42.6)	471 (91.8)	42 (8.2)	
Ethnicity				
Malay	1002 (83.2)	925 (92.3)	77 (7.7)	0.909
Non-Malay	202 (16.8)	186 (92.1)	16 (7.9)	
Marital status				
Married	802 (66.6)	748 (93.3)	54 (6.7)	0.152
Widowed/Divorced	384 (31.9)	346 (90.1)	38 (9.9)	
Never married	18 (1.5)	17 (94.4)	1 (5.6)	
Family composition				
I live alone	64 (5.3)	62 (96.9)	2 (3.1)	0.240 ^b^
I live with my family	1140 (94.7)	1049 (92.0)	91 (8.0)	
Current financial situation				
Difficult	228 (18.9)	195 (85.5)	33 (14.5)	<0.001
Average	796 (66.1)	743 (93.3)	53 (6.7)	
Comfortable	180 (15)	173 (96.1)	7 (3.9)	
Current employment status				
Employed	169 (14.0)	163 (96.4)	6 (3.6)	0.058
Retired from job	868 (72.1)	798 (91.9)	70 (8.1)	
Never had a job	167 (13.9)	150 (89.8)	17 (10.2)	
Body Mass Index (BMI) classification				
Normal	223 (18.5)	206 (92.4)	17 (7.6)	0.379
Underweight	39 (3.2)	36 (92.3)	3 (7.7)	
Pre-obese	454 (37.7)	426 (93.8)	28 (6.2)	
Obese	488 (40.5)	443 (90.8)	45 (9.2)	
Current health status				
Good	712 (59.1)	676 (94.9)	36 (5.1)	<0.001
Fair	441 (36.6)	400 (90.7)	41 (9.3)	
Poor	51 (4.2)	35 (68.6)	16 (31.4)	
Difficulty seeing				
Yes	833 (69.2)	760 (91.2)	73 (8.8)	0.043
No	371 (30.8)	351 (94.6)	20 (5.4)	
Difficulty hearing				
Yes	324 (26.9)	281 (86.7)	43 (13.3)	<0.001
No	880 (73.1)	830 (94.3)	50 (5.7)	
Difficulty walking, climbing steps, and carrying items				
Yes	613 (50.9)	593 (96.7)	74 (3.3)	<0.001
No	591 (49.1)	572 (96.8)	19 (3.2)	
Difficulty remembering or concentrating				
Yes	460 (38.2)	409 (88.9)	51 (11.1)	0.001
No	744 (61.8)	702 (94.4)	42 (5.6)	
Ill/sick in the past 12 months				
No	695 (57.7)	652 (93.8)	43 (6.2)	0.001
Yes, I have been and am still ill/sick	148 (12.3)	126	22	
Yes, I have been but recovered	337 (28.0)	313	24	
I do not remember	24 (2.0)	20	4	
Limitation of activities in the past 6 months				
Severely limited	49 (4.1)	31	18	<0.001
Limited but not severe	445 (37)	385	60	
Not limited at all	710 (59)	695	15	

* median (interquartile (IQ) range), ^a^ chi-square test, ^b^ continuity correction, N/a = not applicable.

**Table 2:** Descriptive and bivariate analysis of activity of daily living (ADL), instrumental activity of daily living (IADL), mobility, and falls associated with the need for assistance.

Variables	Total, *n* (%)	I Do not Need Assistance, *n* (%)	I Need Assistance, *n* (%)	^a^*p*-Value
Bathing				
Independent	1194 (99.2)	1106 (92.6)	88 (7.4)	<0.001 ^b^
Dependent	10 (0.8)	5 (50.0)	5 (50.0)	
Dressing				
Independent	1194 (99.2)	1106 (92.6)	88 (7.4)	<0.001 ^b^
Dependent	10 (0.8)	5 (50.0)	5 (50.0)	
Toileting				
Independent	1195 (99.3)	1108 (92.7)	87 (7.3)	<0.001 ^b^
Dependent	9 (0.7)	3 (33.3)	6 (66.7)	
Transferring				
Independent	1176 (97.7)	1094 (93.0)	82 (7.0)	<0.001 ^b^
Dependent	28 (2.3)	17 (60.7)	11 (39.3)	
Self-control of continence				
Good control	1169 (97.1)	1084 (92.7)	85 (72.7)	0.002 ^b^
Poor control	35 (2.9)	27 (77.1)	8 (22.9)	
Feeding				
Independent	1199 (99.6)	1110 ((92.6)	89 (7.4)	<0.001 ^b^
Dependent	5 (0.4)	1 (20.0)	4 (80.0)	
Able to go out alone by train, bus, or taxi				
Yes	763 (63.4)	725 (95.0)	38 (5.0)	<0.001
No	441 (36.6)	385 (87.3)	55 (12.7)	
Able to go shopping for daily necessities				
Yes	1005 (83.5)	949 (94.4)	56 (5.6)	<0.001
No	199 (16.5)	162 (81.4)	37 (18.6)	
Able to cook				
Yes	1040 (86.4)	970 (93.3)	70 (6.7)	0.001
No	164 (13.6)	141 (86.0)	23 (14.0)	
Able to pay bills				
Yes	858 (71.3)	815 (95.0)	43 (5.0)	<0.001
No	346 (28.7)	296 (85.5)	50 (14.5)	
Able to withdraw or deposit money				
Yes	766 (63.6)	726 (94.8)	40 (5.2)	<0.001
No	438 (36.4)	385 (87.9)	53 (12.1)	
Able to fill out documents				
Yes	692 (57.5)	657 (94.9)	35 (5.1)	<0.001
No	512 (42.5)	454 (88.8)	58 (11.3)	
Able to find friends’ telephone numbers and call them				
Yes	948 (78.7)	886 (93.5)	62 (6.5)	0.003
No	256 (21.3)	225 (87.9)	31 (12.1)	
Able to walk for 15 min without stopping				
Yes	1048 (87)	990 (94.5)	58 (5.5)	<0.001
No	156 (13)	121 (77.6)	35 (22.4)	
Decreased frequency of going out				
Yes	165 (13.7)	141 (85.5)	24 (14.5)	<0.001
No	1039 (86.3)	970 (93.4)	69 (6.6)	
Able to go upstairs without holding on to the handrail or the wall				
Yes	787 (65.4)	752 (95.6)	35 (4.4)	<0.001
No	417 (34.6)	359 (86.1)	58 (13.9)	
Able to stand up from chairs without holding on to anything				
Yes	954 (79.2)	907 (95.1)	47 (4.9)	<0.001
No	250 (20.8)	204 (81.6)	46 (18.4)	
History of falls in the past 1 year				
Many times	70 (5.8)	59 (84.3)	11 (15.7)	<0.001
Once	217 (18.0)	189 (87.1)	28 (12.9)	
No	917 (76.2)	863 (94.1)	54 (5.9)	
Concern about falls				
Yes, very much	522 (43.4)	479 (91.8)	43 (8.2)	0.912
Yes, somewhat	210 (17.4)	195 (92.9)	15 (7.1)	
Only a little	167 (13.9)	157 (94.0)	10 (6.0)	
No	305 (25.3)	280 (91.8)	25 (8.2)	

^a^ chi-square test, ^b^ continuity correction.

**Table 3:** Factors associated with the need for assistance.

Variable	Crude OR (95% CI)	*p*-Value ^d^	Adj OR (95% CI)	*p*-Value ^d^
Current financial situation		<0.001		0.002
Difficult	4.18 (1.80, 9.70)	0.001 ^c^	4.11 (1.67, 10.11)	0.002 ^c^
Average	1.76 (0.79, 3.95)	0.168 ^c^	2.17 (0.92, 5.12)	0.077 ^c^
Comfortable	1.00		1.00	
Current health status		<0.001		0.011
Good	1.00		1.00	
Fair	0.52 (0.33, 0.83)	0.006 ^c^	0.91 (0.55, 1.49)	0.696 ^c^
Poor	4.46 (2.28, 8.74)	<0.001 ^c^	3.15 (1.44, 6.87)	0.004 ^c^
Difficulty hearing				
Yes	2.54 (1.65, 3.90)	<0.001	1.78 (1.11, 2.83)	0.017
No	1.00		1.00	
Difficulty walking, climbing steps, and carrying items				
Yes	4.13 (2.46, 6.94)	<0.001	1.77 (0.99, 3.14)	0.046
No	1.00		1.00	
Limitation of activities in the past 6 months		<0.001		<0.001
Severely limited	26.90 (12.41, 58.33)	<0.001 ^c^	13.31 (5.61, 31.60)	<0.001 ^c^
Limited but not severe	7.22 (4.05, 12.89)	<0.001 ^c^	5.06 (2.72, 9.40)	<0.001 ^c^
Not limited at all	1.00		1.00	

^c^ Wald test; ^d^ likelihood ratio test. Model adjusted for the locality, gender, ethnicity, marital status, family composition, current employment status, BMI classification, difficulty seeing, difficulty remembering or concentrating, and ill/sick in the past 12 months.

**Table 4:** Daily living activities associated with the need for assistance.

Variable	Crude OR (95% CI)	*p*-Value ^d^	Adj OR (95% CI)	*p*-Value ^d^
Feeding				
Independent	1.00		1.00	
Dependent	49.89 (5.52, 451.10)	<0.001	17.03 (1.72, 169.08)	0.005
Able to stand up from chairs without holding on to anything				
Yes	1.00		1.00	
No	4.35 (2.82, 6.72)	<0.001	2.56 (1.56, 4.20)	<0.001
History of falls in the past year		<0.001		0.009
Many times	2.98 (1.48, 6.00)	0.002 ^c^	1.50 (0.69, 3.24)	0.307 ^c^
Once	2.37 (1.46, 3.84)	<0.001 ^c^	2.25 (1.36, 3.74)	0.002 ^c^
No	1.00		1.00	
Able to walk for 15 min without stopping				
Yes	1.00		1.00	
No	4.94 (3.12, 7.82)	<0.001	2.43 (1.43, 4.14)	0.001
Able to pay bills				
Yes	1.00		1.00	
No	3.20 (2.09, 4.92)	<0.001	1.75 (1.07, 2.85)	0.026

^c^ Wald test; ^d^ likelihood ratio test. Model adjusted for bathing; dressing; toileting; transferring; self-control of continence; able to go out alone by train, bus, or taxi; able to go shopping for daily necessities; able to cook; able to withdraw or deposit money; able to fill up documents; able to find friends’ telephone numbers and call them; decreased frequency of going out; able to go upstairs without holding on to the handrail or the wall; and concern about falls.

**Table 5:** Overall factors and daily living activities associated with the need for assistance.

Variable	Crude OR (95% CI)	*p*-Value ^d^	Adj OR (95% CI)	*p*-Value ^d^
Limitation of activities in the past 6 months		<0.001		<0.001
Severely limited	26.90 (12.41, 58.33)	<0.001 ^c^	11.99 (5.04, 28.50)	<0.001 ^c^
Limited but not severe	7.22 (4.05, 12.89)	<0.001 ^c^	4.86 (2.66, 8.87)	<0.001 ^c^
Not limited at all	1.00		1.00	
Difficulty of hearing				
Yes	2.54 (1.65, 3.90)	<0.001	1.78 (1.11, 2.85)	0.018
No	1.00		1.00	
Current financial situation		<0.001		0.01
Difficult	4.18 (1.80, 9.70)	0.001 ^c^	4.56 (1.83, 11.39)	0.001 ^c^
Average	1.76 (0.79, 3.95)	0.168 ^c^	2.36 (0.99, 5.65)	0.053 ^c^
Comfortable	1.00		1.00	
Feeding				
Independent	1.00		1.00	
Dependent	49.89 (5.52, 451.10)	<0.001	8.46 (0.82, 87.15)	0.045
Able to walk for 15 min without stopping				
Yes	1.00		1.00	
No	4.94 (3.12, 7.82)	<0.001	1.99 (1.14, 3.45)	0.017
Able to stand up from chairs without holding on to anything				
Yes	1.00		1.00	
No	4.35 (2.82, 6.72)	<0.001	2.06 (1.25, 3.41)	0.005

^c^ Wald test; ^d^ likelihood ratio test. Model adjusted for the sociodemographic, socioeconomic, and health status variables (locality; age group; gender; ethnicity; marital status; family composition; current employment status; BMI classification; current health status; difficulty seeing; difficulty remembering or concentrating; ill/sick in the past 12 months; bathing; dressing; toileting; transferring; self-control of continence; able to go out alone by train, bus, or taxi; able to go shopping for daily necessities; able to cook; able to withdraw or deposit money; able to pay bills; able to fill out documents; able to find friends’ telephone numbers and call them; decreased frequency of going out; able to go upstairs without holding on to the handrail or the wall; a history of falls in the past year; and concern about falls).

## Data Availability

The data presented in this study are available upon request from the corresponding author.
